# Our future is global: nursing leadership and global health*

**DOI:** 10.1590/1518-8345.4542.3339

**Published:** 2020-08-31

**Authors:** Jane Salvage, Jill White

**Affiliations:** 1 International Council of Nurses, Global Nursing Leadership Institute, Geneva, Switzerland.; 2 The University of Sydney, Susan Wakil School of Nursing and Midwifery, Sydney, Australia.

**Keywords:** Nursing, Leadership, Global Health, Public Health, Health Policy, Sustainable Development, Enfermagem, Liderança, Saúde Global, Saúde Pública, Política de Saúde, Desenvolvimento Sustentável, Enfermería, Liderazgo, Salud Global, Salud Pública, Política de Salud, Desarrollo Sostenible

## Abstract

Global health matters to every nurse everywhere. In this article we outline why. We highlight some important health issues confronting the world today; explore how these issues are being tackled; and consider the implications for nursing. We describe how nurses are making a difference in the challenging contexts, range and complexity of nursing work round the globe, and we conclude with a call to action. Nurses can influence, and become, policy-makers and politicians, and explain to them why the Sustainable Development Goals cannot be reached without strengthening nursing. In this International Year of the Nurse and Midwife, the window of opportunity is open, but it will not stay open for long. Nurses and midwives globally and locally must be ready to jump through it. We ask you to join hands, and join us.

## Abstract (pt)

A saúde global importa para os enfermeiros do mundo todo. Neste artigo, descrevemos o motivo. Destacamos algumas questões de saúde importantes que o mundo está enfrentando atualmente; exploramos como essas questões estão sendo abordadas; e consideramos as implicações para a Enfermagem. Descrevemos como os enfermeiros estão fazendo a diferença nos contextos desafiadores, destacamos a extensão e a complexidade dos trabalhos dos enfermeiros ao redor do mundo, e concluímos com um apelo à ação. Os enfermeiros podem influenciar, e se tornarem, elaboradores de política e políticos, e explicar para eles o motivo pelo qual os Objetivos de Desenvolvimento Sustentável não podem ser alcançados sem fortalecer a Enfermagem. Neste Ano Internacional da Enfermagem e da Obstetrícia, a janela de oportunidade está aberta, mas não ficará aberta por muito tempo. Os enfermeiros e as parteiras devem estar prontos para atravessar essa janela, tanto em nível global quanto local. Pedimos que vocês deem as mãos e juntem-se a nós.

## Abstract (es)

La salud global es importante para cualquier enfermera en cualquier lugar del mundo. En este artículo detallamos por qué. Destacamos algunos problemas de salud importantes que enfrenta el mundo actualmente, estudiamos de qué manera se están abordando estos problemas y consideramos las repercusiones para la Enfermería. Describimos de qué manera las enfermeras están marcando la diferencia en los contextos problemáticos, la amplitud y complejidad del trabajo de Enfermería en todo el planeta y concluimos con una convocatoria para la acción. Las enfermeras pueden influenciar a los encargados de elaborar normas y a los políticos (además de pasar a desempeñar esos roles) y explicarles por qué las Metas de desarrollo sustentable no pueden alcanzarse sin fortalecer la Enfermería. En este Año Internacional de las Enfermeras y Parteras la ventana de las oportunidades está abierta, pero no será así por mucho tiempo. Tanto a nivel global como local, las enfermeras deben estar preparadas para aprovecharla. Les pedimos que se tomen de las manos y que se sumen a nosotros.

## Introduction

As health professionals committed to our local community and country, it is tempting to look no further than our own backyard, where there is always so much about which we care deeply. Yet this solely local focus is not only ostrich-like, but also dangerous: global health is inseparable from local and national health concerns. Infectious diseases, for example, do not recognise borders, and a mingling of germs and genes results in communicable diseases with the potential for rapid global spread. Compare the speed with which the coronavirus and COVID-19 infections spread worldwide, to the four years it took the medieval plague to cross Europe.

The concept of “global health” did not really exist even 20 years ago. Now it embraces a complex concept that engages with all countries and indeed with the health of the planet itself^(^ 1 ^)^. A growing number of governments and organizations are adopting it as a key policy theme. In thinking about nursing today and tomorrow, we must all look beyond our backyards, and understand how what happens in distant places affects the health and health care of our communities, our loved ones, and ourselves - just as what happens in our backyard affects people we will never meet. COVID-19 has surely heightened awareness of this. “Think globally, act locally!”, as the environmental slogan says.

Thinking globally is not an academic exercise but a way of seeing that enriches perspectives, increases knowledge, and makes nurses more motivated and effective as leaders, practitioners, managers, teachers, researchers, policy-makers and activists. It helps us to understand how our work contributes to outcomes not only in health sectors, but also in policy, education, economic relations, and environmental activism.

Nurses have a professional obligation to understand the world in its broader context and base decision-making on an expanded understanding of ourselves, our patients, and our circumstances. “It begins with understanding the policies and politics of globalization, the growing interdependence of the world’s people, [which] means that national policy and action are increasingly shaped by international forces along with other aspects of our lives”^(^ 2 ^)^. We increasingly rely on the same groups of workers and technologies and face the same environmental and epidemiological threats. Moreover, the policies that most affect health are not always health policies^(^ 3 ^)^. Other policies have enormous impact on health determinants and solutions, so cross-sectoral collaboration on global health is critical.

### The wealth of a nation is its people - and their health

We live in challenging times, for the health of the planet, nations and communities. The challenges have major implications for nursing as a global profession of some 23 million, from halting pandemics to reducing mother and child deaths, tackling and mitigating the effects of climate change, and caring for older people. Inequalities between groups of people, and within and between countries and regions, are key to understanding these challenges.

There is a mass of evidence of the interaction between health and wealth at all levels, whether individual, family, community or country. Nurses know that their patients’ ways of life and the conditions in which they live and work strongly influence their health and longevity. This challenges us to complement biomedical models of health care with social models, and focus much more on prevention and public health.

Since the 1990s there has been a growing understanding of the interaction between health and poverty, and the need for cooperation and collaboration on a global scale to combat its consequences for health and for economies. This has stimulated many organizations to play a larger role in global health. Eradicating poverty in all its forms and dimensions is the greatest global challenge, says the United Nations: “bold and transformative steps [...] are urgently needed to shift the world onto a sustainable and resilient path”^(^ 4 ^)^.

The action framework for these “bold and transformative steps” for all countries, irrespective of development or resource level, is the 17 Sustainable Development Goals (SDGs) for 2016-2030. They are inextricably interwoven. Health is inherent in all goals and influenced by all goals, and there is one overt health goal. They provide an overarching global framework for health policy and practice, and drive the work of the World Health Organization (WHO) and other global organisations.

In fact there are thousands of organizations of all types, sizes and ambitions active in global health, making it a highly complex, often competitive and politically contested terrain. Where is nursing in this landscape? The profession has a much longer global presence than the UN or WHO: the International Council of Nurses (ICN) was founded in 1899, predating WHO by nearly 50 years, and funded the first WHO nursing post. ICN has three primary functions: to represent nursing worldwide, to advance the profession, and to influence health policy. It remains the major global voice of nursing, though its ambitions are hindered by lack of funds, and the difficulties of modernising and leading with such a diverse group of member associations. It has recently adopted a more overtly collaborative, interprofessional and intersectoral approach, seeking greater influence on health and health care to complement its traditional focus on advancement of the profession. This means wider engagement with other organizations, moving outside the “nurses’ house” in which nurses have lived for over a century^(^ 5 ^)^.

Many more nursing organisations and networks are active in global health, including the global network of WHO Collaborating Centres in Nursing and Midwifery, and those active on specific nursing concerns and specialities ranging from primary health care to cancer nursing to climate change. Nongovernmental organizations, health services and universities throughout the world also have a rich diversity of intercountry bilateral and multilateral relationships and projects that contribute to the global health agenda. Thousands of nurses meet regularly at international congresses across the globe, sharing ideas and research.

Nursing, however, is seriously underrepresented in the major global health organizations, while nurse-led organisations are comparatively weak and lacking in influence, and are not major players. These shortcomings, which have a large and generally unrecognised negative impact on global health, are part and parcel of structural global inequalities related to gender, wealth, race, status and other issues. The lack of a strong global nursing voice is one manifestation of this, alongside the lack of strong voices from low-income countries, indigenous peoples and other disadvantaged groups. Sadly, the coronavirus pandemic has demonstrated once again the silence around nursing: media coverage of expert opinions, and of deaths among health workers, has focused almost entirely on physicians, rather than the nurses who give 24-hour care and die in far larger numbers.

### Global health politics and policy

Many nurses make naive assumptions about health and healthcare and do not view these issues through a sociopolitical lens^(^ 6 ^)^. Yet we need to understand some history and politics if we want to be leaders of change, rather than its servants.

The market-based, “neoliberal” political and social philosophy that swept the world in the 1980s, and continues to dominate it, has a profound effect on global health and global health politics. The powerful influence of vested commercial interests cannot be underestimated, including the groups of huge corporations known as Big Tobacco, Big Sugar and Big Pharma. Action on climate change is another policy battleground: the muted and poorly coordinated response of governments to climate change, and the science explaining it, is a further example of the intensely political nature of global health policy, strongly influenced by national politics.

Political arguments along party lines in countries have far-reaching and serious health consequences. National changes in political philosophy can quickly change the global health funding environment. The politics of health is fraught with vested interests and contested priorities, and at global level this complexity expands exponentially. The big question is what impact all this activity has - how far it builds capacity and is truly developmental and sustainable. The success rate of international projects is surprisingly low and some do more harm than good, which has led to growing criticism of the global health establishment, and the rise of grass-roots social movements like the People’s Health Movement (PHM). This global network, committed to addressing the social, environmental and economic determinants of health, brings together health activists, civil society organizations and academic institutions, particularly from low and middle income countries. “The world is facing a global health crisis characterized by growing inequities within and among nations and millions of preventable deaths, especially among the poor” it says - largely due to unfair economic structures which lock people into poverty and poor health^(^ 7 ^)^.

### Policy, politics and nursing

Policy and politics determines not only the health of populations but also nursing itself - past, present and future. It profoundly shapes the practice and workplaces of nurses at local, regional, national and international levels. Nurses who wish to influence and lead policy, rather than be bystanders, should understand not only the content related to a health issue, but also the policy process, the context, and the stakeholders and their interests^(^ 8 ^)^.

The largest proportion of the health workforce globally by a large margin, nurses are often the only health care provider available. We are key to ensuring that all people and communities receive the health services they need without financial hardship. Nurses occupy a special position as the interface between the health system and the community; we see, hear and know, as end users of health policies, how policy affects people and their communities. You might think that knowledge would be welcomed with open arms by policy-makers, yet it has been very difficult for nurses at all levels to make an impact on policy, for a variety of reasons^(^ 3 ^)^. While nurses are acknowledged as key policy implementers - the pairs of hands, they are rarely central to health and social policy development - at the top table^(^ 6 ^)^.

Nurses engaged in high-level global health work apply their nursing lens to issues that others may not notice. They bring information from the field to high-level meetings, explain the complexities of implementing programmes, and interpret the science or recommendations from these meetings back to the field in a way that may be translated into action. Australian nurse Amanda McClelland, for example, was the senior officer in the emergency health unit of the International Federation of Red Cross and Red Crescent Societies. “I added a social mobilisation and community aspect to global strategy discussions”, she said^(^ 9 ^)^. “How am I going to explain this to the volunteers and how will they explain it to the community? That’s great, but the community would never accept it. That’s great, but we won’t be able to implement the programme in that way. We’re going to need to consider weather/culture/religious factors when rolling this out.”

Nurses worldwide have become increasingly knowledgeable, skilled and well educated, like McClelland, but this has not been matched with a significant growth in their influence and status. The exclusion of senior nurses from leadership positions in health organizations at all levels, and even the very existence of leadership positions within nursing, are critical factors. The profession often challenges this, and sometimes makes headway, but has to fight the battle all over again when health employers decide they no longer need a nurse director, or when governments do not replace their chief nurse. Even in countries that have traditionally provided global nursing leadership, the government chief nurse role has been abolished, downgraded, or never existed. Even in WHO, now reinventing itself as a champion of nursing and midwifery, the chief nurse and head nurses in the six WHO regional offices used to have much larger teams and budgets. All have declined in scope and influence, attributable partly to cuts in WHO budgets, but also to long-standing reluctance to recognise the value of the nursing contribution.

### Understanding policy-making

Getting a seat at the top table is one thing, being effective once there is another. Nurses who occupy senior positions may not be influential, for a range of reasons ranging from gender discrimination to lack of status. Many have little or no preparation for these roles and do not know how to influence and shape policy. Moreover, “political space is finite […]. Organised medicine knows when and how to present a united front [...]. Nursing is far less politically accomplished and far less assertive”^(^ 10 ^)^.

Speaking with one voice and in a language that appeals to policy-makers has not been one of nursing’s successes^(^ 6 ^)^. Nor has the ability to persuade policy-makers to take effective action on nursing issues. Nursing history in many countries and at regional and international levels is strewn with evidence-based policy reviews and reports making excellent recommendations that go largely unheeded. Compounded by structural inequalities related to gender and social class, nurses’ attempts to push for reform have not gained enough traction, and change has not happened fast enough or far enough.

Policy leadership is required in the everyday workings of national and local government and health systems. A key nursing competency, it is rarely acknowledged or formally developed; most nurses learn it, if at all, by bitter experience. The need for nurses to develop these competencies has long been highlighted^(^ 11 ^)^, but not consistently developed across the global nursing community. White advocates “policy leadership and role modelling” by nurse leaders, who need the right professional, political and policy skills to operate effectively in tough arenas. Whether they work in government, management, education, advanced practice, research or development, they need to know how to maximize their distinctive contribution to shaping, influencing and implementing policy decisions^(^ 12 ^)^. This requires nurses to grasp White’s concept of a “new pattern of knowing called socio-political knowing”^(^ 6 ^)^ and, as Salvage has long advocated, to become policy activists who are politically savvy^(^ 13 ^)^.

### The window of opportunity

Reports and recommendations on nursing that fail to have traction, policies that ignore or undermine nursing, and nurses’ absence from policy-making - this gloomy pattern is starting to change for the better. More nurses are becoming policy entrepreneurs: leaders who position themselves to influence policy; who bring together problems, policies and politics into a novel amalgam - new policy; and who soften up the system by presenting participants in the network (visible and invisible) with alternative representations of their realities. This leads to the opening of a window of opportunity, as described by Kingdon - the potential for a truly new policy perspective^(^ 14 ^)^.

That window is opening wider as demand grows worldwide for solutions to acute problems including current and future health worker shortages, and the rising need for expert care of older people, alongside huge public interest in nursing. There is greater global awareness of the importance of investment in health as a public good, and of nurses’ massive actual and potential contribution to improving health, creating gender equality and strengthening economies. Meanwhile more nurses are finding the courage to become “silence breakers” and join the worldwide wave of protests against violence, sexual harassment and other abusive behaviour against women^(^ 15 ^)^.

The window was pushed a little further open by a call for action in an influential UK report that saw how national and global issues are interconnected, and explicitly highlighted the social and economic impact of nursing^(^ 3 ^)^. Advocating greater investment in nursing to bring rich returns and rewards for global health, it highlighted the “triple impact” of nursing worldwide, namely better health, greater gender equality, and stronger economies. Building on the positive impact of these new ways of thinking about nursing, its recommendations were taken up by the Nursing Now campaign, in partnership with ICN and WHO. Dr Tedros Adhanom Ghebreyesus, the first ever WHO Director-General who is not a physician, appointed a Chief Nursing Officer in 2017, Elizabeth Iro - the first time in its 70-year history that a nurse has sat at WHO’s top table. In 2020 WHO also produced its first ever *State of the World’s Nursing* report, an important step - but the fact that it took WHO 70 years to get round to it is a measure of its historical failure to have nurses and nursing at its top tables and high up its agendas.

### A new story of nursing

The major shifts necessary to transform nursing will not be effected through a continuing series of short-term, piecemeal policy initiatives, however good each may be. Deep-rooted, sustainable change will depend on reaching honest, shared understanding of the barriers to change and the structural inequalities and issues that maintain them, and on tackling the root causes and underlying drivers.

These big issues are not solved by tips on how to exploit the status quo, and patience is unlikely to be the answer either. This is the moment for nurses to shift the paradigm, to be taken seriously together and individually, when the old certainties and ways are being shaken to the core. Nursing organizations, as well as trying to gain influence at the top tables, are making alliances with social movements and considering radical alternatives.

All this could lead us to a new story of health and healthcare^(^ 3 ^)^. Nurses, as leading actors in this story, will be at the heart of sustainable health systems that meet individual and population needs, are fit for the present, and innovative and adaptable for the future. Rooted in reality, yet reaching for the stars, nurses work to shape sustainable, high quality, effective and affordable services fit for the future, and responsive to the challenges of turbulent times. They focus on where the needs are greatest and where there is most potential to gain health and reduce inequalities. They take their understanding and experience as hands-on practitioners into all their subsequent roles, as clinicians, managers, teachers, researchers, scholars, policy-makers and leaders. At all levels, from ward to board to international organizations, they inspire and lead.

We began by arguing that ‘doing global health’ means thinking globally and acting locally: adopting a mindset that seeks to understand the structural and political conditions that sustain armed conflict, poverty, inequality, inaction on environmental pollution, and the poorer health and well-being of vulnerable populations. This does not have to mean travelling beyond your backyard. It may mean working with a local disadvantaged and vulnerable community. It may mean political action to achieve clean air and water. It may mean policy pressure to achieve social justice and universal health coverage. See Box 1 for suggestions on what you could do (adapted from^(^ 2 ^)^). “Thinking globally and acting locally” is old wisdom, but never more needed of nurses and nursing than now.

**Box 1:** How nurses can engage with global health

Begin at home - think globally and act locally.

Cultivate a worldview; be sensitive to the cultural aspects of policy and practice.

Commit to learning more about the global health agenda, above all the SDGs.

Know where regional and international organizations and your national and local government stand on key international health and nursing matters, and lobby them.

Get involved in global health issues, and team up with like-minded groups and people at home and internationally.

Through your professional association, trade union, workplace or community, help colleagues in and from other countries - and learn from them - as they work to strengthen nursing and health.

Advocate, initiate, and document nursing's role in policy.

Join others in ensuring that national and local structures and roles are in place so that nurses' voices are heard in policy and practice.

Ensure that nursing leaders - and new nursing graduates - know about policy and politics, how to analyze the environment, how to develop strategy, and how to work together.

Undertake and disseminate research to build evidence of nursing effectiveness.

Share your ideas and achievements through discussions, publications, conferences, social media and the Internet.

We have also argued that the challenges facing the planet and our own communities have major implications for nursing and nurses, and shared our hope that nurses can influence (and become) politicians and policy-makers, showing them that the SDGs cannot be reached without strengthening nursing. In this Year of the Nurse and Midwife, the window of opportunity is open, but it will not stay open for long. Nurses and midwives globally and locally must be ready to jump through it. Will you join hands, and join us?
